# An elemental approach to modelling the mechanics of the cochlea

**DOI:** 10.1016/j.heares.2017.10.013

**Published:** 2018-03

**Authors:** Stephen J. Elliott, Guangjian Ni

**Affiliations:** a Institute of Sound and Vibration Research, University of Southampton, Southampton, SO17 1BJ, UK; b Lab of Neural Engineering & Rehabilitation, Department of Biomedical Engineering, College of Precision Instruments and Optoelectronics Engineering, Tianjin University, Tianjin, 300072, China; c Tianjin International Joint Research Center for Neural Engineering, Academy of Medical Engineering and Translational Medicine, Tianjin University, Tianjin, 300072, China

**Keywords:** Cochlea, Basilar membrane motion, Micromechanics, Fluid coupling, Elemental model, BM, basilar membrane, CF, characteristic frequency, OHC, outer hair cell, SM, scala media, SV, scala vestibuli, ST, scala tympani, 1D, one-dimensional, 3D, three-dimensional, Dof, degree of freedom

## Abstract

The motion along the basilar membrane in the cochlea is due to the interaction between the micromechanical behaviour of the organ of Corti and the fluid movement in the scalae. By dividing the length of the cochlea into a finite number of elements and assuming a given radial distribution of the basilar membrane motion for each element, a set of equations can be separately derived for the micromechanics and for the fluid coupling. These equations can then be combined, using matrix methods, to give the fully coupled response. This elemental approach reduces to the classical transmission line model if the micromechanics are assumed to be locally-reacting and the fluid coupling is assumed to be entirely one-dimensional, but is also valid without these assumptions. The elemental model is most easily formulated in the frequency domain, assuming quasi-linear behaviour, but a time domain formulation, using state space method, can readily incorporate local nonlinearities in the micromechanics. Examples of programs are included for the elemental model of a human cochlea that can be readily modified for other species.

## Highlights

• General formulation of an elemental model for cochlear mechanics.
• Reduce to the transmission line model for locally-reacting micromechanical and 1D fluid coupling.
• Incorporation of non-uniform areas, 3D fluid coupling and non locally-reacting micromechanics.
• MATLAB programs for the elemental model in the frequency domain and time domain.

## 1 Introduction: modelling the mechanics of the cochlea

It is important to develop mathematical models of the mechanics of the cochlea in order to test our understanding of the physical processes involved in hearing. These models can have various levels of abstraction, depending on what features of the cochlear mechanics they are attempting to describe. Wave models, for example, are good at describing the global behaviour of the motion along the cochlea without getting too involved in the detailed physical processes that give rise to the wave motion. The most widely used wave model uses the WKB method (de Boer and Viergever, 1982, Steele and Taber, 1979a, Steele and Taber, 1979b, Wang et al., 2016), in which the response along the cochlea can be predicted from an assumed distribution of complex wavenumbers. There are, however, several assumptions inherent in using the WKB method. Although it can account for both forward and backward travelling waves in the cochlea, its main assumption is that there is only one type of wave propagating. This is generally called the “slow wave”, to distinguish it from the so-called “fast wave” that travels at the compressional speed of sound in the cochlear fluids. The fast wave is not thought to play an important role in normal hearing, since the pressure is the same in the two fluid chambers and so there is no pressure difference acting on the basilar membrane, BM, which is then not driven into motion. There are, however, several other kinds of wave that might play a significant role in determining the mechanical response of the cochlea, including those due to higher-order fluid modes (Watts, 2000, Elliott et al., 2013) and those due to mechanical coupling along various parts of the organ of Corti or the fluid within it (Zwislocki and Kletsky, 1979, Ghaffari et al., 2007, Ramamoorthy et al., 2007, Lamb and Chadwick, 2011, Meaud and Grosh, 2010). The way that these and other modes couple into the slow wave is still a matter of some debate.

An alternative approach to modelling the mechanics of the cochlea is to divide the length of the cochlea into a finite number of elements and describe the individual physical processes involved in cochlear mechanics. These equations can be coupled together in a numerical model that makes no explicit assumptions about the type of wave propagation. The earliest example of this approach is the “transmission line” model (Shera and Zweig, 1991, Zweig, 1991, Zwislocki, 1950), in which the inertance of the fluid in the cochlear chambers is represented as a series inductance and the response of the BM is represented as a shunt impedance. At the other extreme, the detailed behaviour of the fluid in the chambers and the motion of the organ of Corti could be represented by a finite element model, where both the fluid in the chambers and the different parts of the organ of Corti are meshed into many small elements and the coupled set of equations of motion are, typically, solved using commercial software such as ANSYS (Ni et al., 2016). It is important to note that such finite element methods simultaneously solve for both the pressure in the fluid and the motion of the organ of Corti. This can significantly increase the computational time compared to solving for the fluid pressure and the organ of Corti motion individually, since the computational time typically rises in approximate proportion to the square of the total number of degree-of-freedom in a finite element model. It also means that both the fluid coupling and the organ of Corti motion must be analysed numerically, even if there may be a simple analytic formulation in one case or the other.

An elemental approach to the formulation of cochlear mechanics can be viewed as a generalisation of the transmission line model, in that the fluid motion and the organ of Corti dynamics are analysed separately and then coupled together. In the elemental model, however, the organ of Corti dynamics are not restricted to being locally-reacting and the fluid flow is not restricted to being proportional to the pressure difference between adjacent elements. The elemental model reduces to a transmission line model if these restrictions are imposed, but can also account for the more general case.

This paper first introduces the elemental model, for the case of a locally-reacting basilar membrane and 1D fluid coupling in a uniform box model. If the BM response is linear, the coupled solution can be solved efficiently in the frequency domain and this formulation is considered first. A time domain formulation is considered at the end of this paper which leads to a state space implementation of the elemental model that can be used to simulate the non-linear response of the cochlea but is also important in assessing the stability of linear models. It is shown how the fluid coupling part of the formulation can be adapted to model non-uniform distributions of fluid chamber area and the near field component of the pressure that is present when the fluid coupling is analysed in 3D. More general forms of the BM response are also then considered, where both longitudinal mechanical coupling along the BM and non-symmetrical feedforward behaviour along the organ of Corti can be accounted for.

The theoretical formulation behind the fluid coupling within the cochlea and its passive micromechanics are reasonably well understood. The intention of the present review paper is to demonstrate how these individual responses may be integrated into a numerical formulation that can be readily used to predict the coupled response of the cochlea under different conditions. Although there have been a number of micromechanical models proposed for the active cochlea, there is still considerable debate about the form of such a model and how it should couple into the fluid. Since this is still unclear, and also for reasons of brevity, the elemental model will be developed and illustrated here only using passive micromechanical models, on the understanding that active models can be incorporated with a more general form of BM admittance.

## 2 Formulation of the elemental model in the frequency domain

**Fig. 1:**
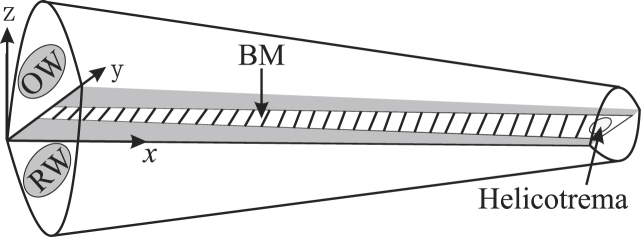
The uncoiled elemental model of the cochlea.

In this section, we assume a linear micromechanical response, and an excitation of the cochlea at a single frequency, so that the amplitude and phase of all the dynamic variables can be considered complex and proportional to *e*^*iωt*^. The essence of the elemental model is that the length of the cochlea is divided into a finite number of sections and that the interaction between the fluid in the chambers and the mechanical response of the BM is described in terms of the transverse modes of the element in each section. The general form of the assumed model is illustrated in Fig. 1, which also shows the coordinate system that will be used below. The cochlea is shown uncoiled since the coiling is not thought to play an important role in its dynamics (Steele and Zais, 1985). The origin of the coordinates system is on the basilar membrane at the base of the cochlea and it consists of a longitudinal variable, *x,* a radial variable, *y*, and a transverse variable, *z*. The aim of the elemental model is to reduce the complicated interaction that occurs between the fluid and the motion of the BM in all three directions to a single set of discrete equations, as a function of only the longitudinal variable, *x*.

### 2.1 Frequency domain formulation

In general, there may be multiple transverse modes across the basilar membrane, but in practice the fluid coupling is relatively insensitive to the exact form of the radial BM velocity distribution (Ni and Elliott, 2013) and so only a single transverse mode is assumed. If the normalised shape of this transverse mode is *ψ*(*y*) and *v*(*x*, *y*) is the complex BM velocity distribution in the longitudinal and radial directions at a frequency *ω*, the dependence of which is suppressed for notational convenience, then the single variable can be used to express the complex BM velocity at the longitudinal position *x*, as defined as

(1) $$v\left(x\right)=\frac{1}{W}\int_{0}^{W}\psi \left(y\right)v\left(x,y\right)dy\text{,}$$

where *W* is the width of the cochlear partition and the normalisation of the mode shape is assumed to be such that

(2) $$\frac{1}{W}\int_{0}^{W}\psi^{2}\left(y\right)dy=1.$$

In a similar way, a single variable describing the difference in pressures in the two main fluid chambers that act on the element is defined to be

(3) $$p\left(x\right)=\frac{1}{W}\int_{0}^{W}\psi \left(y\right)\left[p_{1}\left(x,y,0\right)-p_{2}\left(x,y,0\right)\right]dy\text{,}$$

where *p*_1_(*x*, *y*, *z*) and *p*_2_(*x*, *y*, *z*) are the three-dimensional pressure distributions in the upper and lower fluid chambers.

The continuous distribution of BM velocity and pressure, *v*(*x*) and *p*(*x*), are now spatially sampled to give a finite set of BM velocities and pressures at a number of discrete points, *v*_*n*_ and *p*_*n*_, where the integer variable, *n*, runs from 2 to *N*. The first element in the set of velocities and pressures, *n* = 1, in these arrays is reserved to describe the excitation at the oval window from the middle ear, and the final element, *n* = *N*, is used to define the conditions at the helicotrema. Although it is not essential to the formulation, we assume here that the spatial sampling is uniform, so that if the length of the cochlear is *L* then the length of the elements in the longitudinal direction is always equal to *L*/*N*, which is defined as Δ. The number of elements and hence their length is determined by the shortest wavelength that needs to be reproduced in the longitudinal direction. A rule of thumb commonly used in finite element analysis is that they should be at least six elements within the shortest wavelength, so in practice it is common to use about 500 elements to describe the wave motion, which may have a wavelength as small as 0.5 mm (Shera, 2007), in a human cochlea of length 35 mm.

Vectors of these discrete spatial samples of complex pressure and BM velocity are now defined as

(4) $$\begin{array}{l} p={\left[p\left(1\right),p\left(2\right),\cdots p\left(N\right)\right]}^{\text{T}}\\ v={\left[v\left(1\right),v\left(2\right),\cdots v\left(N\right)\right]}^{\text{T}}\text{,} \end{array}$$

which are coupled both because of the fluid in the chambers and also because of the micromechanical response of the BM. The fluid coupling in the chambers is now assumed to generate a set of pressures, **p**, equal to a fluid coupling matrix, **Z**_FC_, multiplied by the vector of elemental velocities **v**,

(5) $$p=Z_{\text{FC}}v\text{.}$$

The vector of BM velocities, **v**, is also assumed to be equal to the sum of an excitation vector, which contains the imposed stapes velocity, **v**_s_, minus the matrix of BM admittance, **Y**_BM_, multiplied by the pressure vector, **p**,

(6) $$v=v_{\text{s}}-Y_{\text{BM}}p\text{.}$$

The negative sign in equation (6) accounts for the fact that in our convention a positive pressure difference drives the basilar membrane in a downward direction, whereas the elements of the vector **v** are defined to be those in an upward direction. Normally all the elements of the excitation vector **v**_s_ are taken to be zero except the first element, which is equal to the normalised stapes velocity. More generally, it is also possible to represent internal velocity excitation of the elements along the basilar membrane by setting other elements of **v**_s_ to be equal to this internal excitation. This can be used, for example, to model the generation of DPOAEs (Kanis and de Boer, 1997, Young et al., 2012).

By combining equations (5), (6) and assuming that the matrix [**I** + **Y**_BM_**Z**_FC_] is not singular, an explicit expression can be derived for the vector of complex velocities along the basilar membrane as a function of the basilar membrane admittance matrix, the fluid coupling impedance matrix and the excitation vector as

(7) $$v={\left[I+Y_{\text{BM}}Z_{\text{FC}}\right]}^{-1}v_{\text{s}}\text{.}$$

This formula is the same as that used to solve similar coupled fluid-structural interaction problems in engineering, when they are formulated in elemental terms (see, for example, Fahy and Gardonio, 2007).

Substituting equation (7) into equation (5), the expression for the vector of complex pressures is also obtained as

(8) $$p=Z_{\text{FC}}{\left[I+Y_{\text{BM}}Z_{\text{FC}}\right]}^{-1}v_{\text{s}}\text{,}$$

using the matrix identity **A**^−1^**B**^−1^ = [**BA**]^−1^, where in this case **A** and **B** are equal to $Z_{\text{FC}}^{-1}$ and [**I** + **Y**_BM_**Z**_FC_], the vector of pressures can also be written as

(9) $$p={\left[Z_{\text{FC}}^{-1}+Y_{\text{BM}}\right]}^{-1}v_{\text{s}}\text{,}$$

which will be used below.

### 2.2 1D fluid dynamics

The cross-sectional area of the two fluid chambers is initially assumed to be constant along the cochlea, as in the uniform box model (de Boer, 1996). If the pressure in each of the fluid chambers is assumed to be uniform over each cross-section, then the fluid dynamics can be described as being 1D. Physically, the condition under which it is valid to use 1D fluid dynamics is when the shortest wavelength of the slow wave in the cochlea is greater than the cross-sectional dimensions of the fluid chamber. This condition is not a bad approximation in the passive cochlea, but becomes less valid when the cochlea is active and the shortest wavelength becomes smaller, in which case a 3D description of the fluid dynamics, as discussed below, should be used.

Assuming 1D fluid coupling, the equation for the fluid mass and momentum conservation can be combined to provide a differential equation relating the longitudinal distribution of the complex pressure difference across the BM, *p*(*x*), to that of the complex BM velocity distribution, *v*(*x*), both proportional to *e*^*iωt*^, as

(10) $$\frac{\partial^{2}p\left(x\right)}{\partial x^{2}}=-\frac{2i\omega \rho }{h}v\left(x\right)\text{,}$$

where *h* is the effective height of the fluid chambers, given by *π*^2^*A*/8*B*, where *A* is the area of each chamber and *B* is the width of the BM, as discussed below. Assuming that the wavelength of the slow wave is small compared with the length of the elements, then the second spatial derivative in equation (10) can be approximated using a finite difference approach as

(11) $$\frac{p_{n-1}-2p_{n}+p_{n+1}}{\Delta^{2}}=-\frac{2i\omega \rho }{h}v\left(n\right)\text{.}$$

The boundary condition at the base of the cochlea can be written in terms of the momentum equation for the complex pressure as

(12) $$\frac{\partial p\left(x\right)}{\partial x}{|}_{x=0}=-2i\omega \rho v_{1}\text{,}$$

where *v*_1_ is the stapes velocity when *p*_1_ is equal to 0, i.e. when the middle ear in unloaded, as described below. Using the finite difference approximation for the spatial derivative in equation (12), we obtain

(13) $$\frac{p_{2}-p_{1}}{\Delta }=-2i\omega \rho v_{1}\text{.}$$

The boundary condition at the apex of the cochlea, at the helicotrema, is generally taken to be pressure release, i.e. the pressure difference across the BM is equalised in the two fluid chambers so that *p*_N_ = 0.

These finite difference equations can be written in matrix form as

(14) $$i\omega \left[\begin{array}{c} v_{1}\\ v_{2}\\ \vdots \\ v_{N-1}\\ v_{N} \end{array}\right]=\frac{h}{-2\rho \Delta^{2}}\left[\begin{array}{cccccc} -\frac{\Delta }{h} & \frac{\Delta }{h} & & & & \\ 1 & -2 & 1 & & & \\ 0 & 1 & -2 & 1 & & \\ & & \ddots & & & \\ & & & 1 & -2 & 1\\ & & & 0 & 0 & \frac{\Delta^{2}}{h^{2}} \end{array}\right]\left[\begin{array}{c} p_{1}\\ p_{2}\\ \vdots \\ p_{N-1}\\ p_{N} \end{array}\right]\text{,}$$

or more compactly in terms of the vectors of velocity and pressure as

(15) iωv=Fp,

where **F** is the matrix of finite difference terms above. Assuming that this matrix is not singular then we can write the vector of pressures as

(16) $$p=i\omega F^{-1}v\text{,}$$

which is of the form assumed above for the fluid dynamics with

(17) $$Z_{\text{FC}}=i\omega F^{-1}\text{.}$$

The need to be able to invert this matrix requires that a finite value be entered into its bottom right element, taken here as *Δ*^2^/*h*^2^ but discussed more fully below.

If the vector of complex pressures is written in the form of equation (9), then using equation (16) this vector can be written as

(18) $$p={\left[\frac{1}{i\omega }F+Y_{\text{BM}}\right]}^{-1}v_{\text{s}}\text{,}$$

which is of the form originally used by Neely (1981), so that

(19) $$v_{\text{s}}=\left[\frac{1}{i\omega }F+Y_{\text{BM}}\right]p\text{,}$$

The final, *N*-th, element of **v**_s_ is equal to the inner product of the bottom row of the matrix [1/*iω***F** + **Y**_BM_] and the vector **p**. Since all the elements in the bottom row of **Y**_BM_ are zero and only the final element in the bottom row of **F** is finite, then the final element of **p**, *p*(*N*), is proportional to the final element of **v**_s_, *v*_s_(*N*), which is set to zero, thus imposing the helicotrema boundary condition.

### 2.3 Locally-reacting micromechanics

The mechanical coupling along the length of the BM is relatively weak and longitudinal coupling in the cochlea is dominated by that due to the fluid in the chamber, so that a reasonable first approximation is to assume that the BM is locally reacting. The BM is said to be locally reacting if the velocity of an element at one position depends only on the pressure difference at that point, so that

(20) $$v_{n}=-Y_{\text{BM}}\left(n\right)p_{n}\text{,}$$

where *Y*_BM_(*n*) is the mechanical admittance of the BM and the negative sign has been discussed above. If the passive BM dynamics are approximated by a single degree of freedom system, then

(21) $$Y_{\text{BM}}\left(n\right)=\frac{i\omega }{i\omega r_{n}-\omega^{2}m_{n}+s_{n}}\text{,}$$

where *ω* is the excitation frequency and *m*_*n*_, *s*_*n*_ and *r*_*n*_ are the mass, stiffness and damping, per unit area, of the BM at the position of the *n*-th element, i.e. at a distance of *x*_*n*_ = *n*Δ along the cochlea.

It is assumed in the example below that BM mass, *m*_*n*_, is constant along the cochlea, and is denoted, *m*_0_. The BM stiffness is chosen to match the distribution of natural frequencies along the cochlea, which is assumed to take the form

(22) $$f\left(x\right)=f_{\text{B}}e^{-x/l}\text{,}$$

where *f*_B_ is the natural frequency at the base and *l* is a characteristic length for the frequency variation, so that

(23) $$s_{n}={\left[2\pi f\left(n\Delta x\right)\right]}^{2}m_{0}\text{.}$$

The BM damping is chosen to ensure that the *Q* factor of the micromechanical elements is constant along the cochlea (de Boer, 1996), and equal to *Q*_0_, so that

(24) $$r_{n}=\frac{\sqrt{s_{n}m_{0}}}{Q_{0}}\text{.}$$

To account for the mechanical loading of the middle ear by the cochlear input impedance we can write the velocity of the first element as

(25) $$v_{1}=v_{\text{st}}-Y_{\text{ME}}p_{1}\text{,}$$

where *v*_st_ is the unloaded stapes velocity and *Y*_ME_ is the admittance looking into the middle ear from the cochlea. This admittance corresponds to the reciprocal of the parameter M_3_ in the analysis of Puria (2003), who shows that this admittance is well approximated by that of a single degree-of-freedom system and who gives appropriate values for the mass, stiffness and damping per unit area in this case.

### 2.4 Transmission line model

**Fig. 2:**
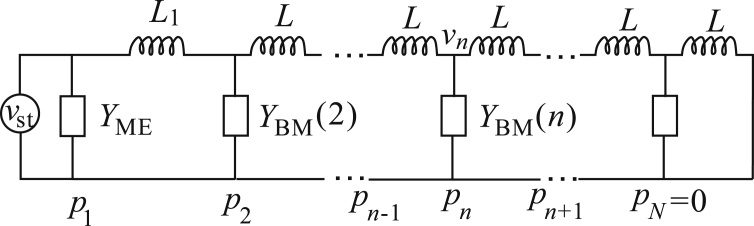
Transmission line interpretation of the elemental model in the particular case of 1D fluid coupling and locally-reacting micromechanics.

**Fig. 3:**
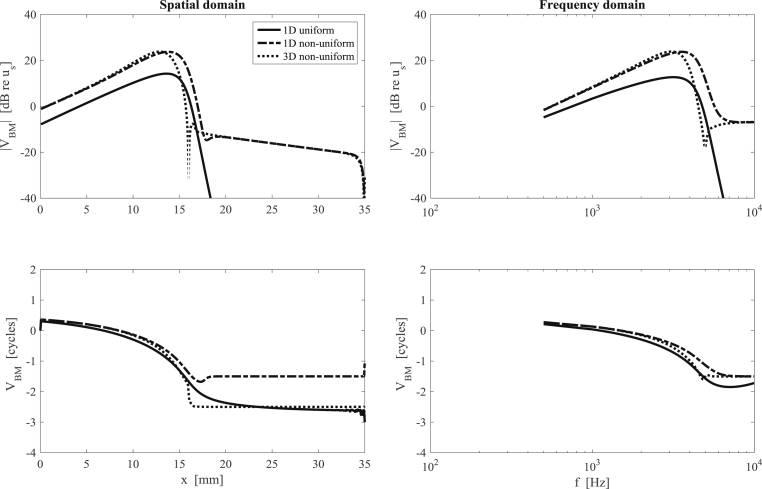
The BM response calculated using the elemental model of the human cochlea in the spatial domain (left column) and in the frequency domain (right column) with either a uniform scala area and 1D fluid coupling (solid line), non-uniform scala area and 1D fluid coupling (dot-dashed line) or non-uniform scala area and 3D fluid coupling (dashed line).

For the special case of 1D fluid coupling and locally-reacting micromechanics, we show that the elemental model can be interpreted in terms of a transmission line, as shown in Fig. 2. The BM velocity is the analogue of current in this network, and pressure difference is the analogue of the voltage. The velocity into the *n*-th shunt element, shown in Fig. 3, where *n* is greater than one, is given by

(26) $$-v_{n}=\frac{p_{n-1}-p_{n}}{i\omega L}+\frac{p_{n+1}-p_{n}}{i\omega L}\text{,}$$

where *L* is the inertance of the longitudinal coupling elements, *p*_*n*_ is the pressure at the *n*-th elements and *v*_*n*_ denotes the BM velocity in the upward direction. This equation can also be written as

(27) $$\frac{p_{n-1}-2p_{n}+p_{n+1}}{i\omega L}=-v\left(n\right)\text{.}$$

By comparing this with equation (11) we find that the series inertance due to the 1D fluid coupling is generally equal to

(28) $$L=2\rho \Delta^{2}/h\text{.}$$

The exception to this is the first series element, which can be seen from equation (12) to have the value

(29) $$L_{1}=2\rho \Delta \text{.}$$

The velocity of the *n*-th elements, for *n* greater than one, is equal to the BM admittance multiplied by the pressure at this point, as expressed in equation (20). The velocity into the first element is given by equation (25), so that the source term at the stapes is indicated as a current source in Fig. 2 and *Y*_ME_ is the first shunt element. The transmission line is terminated by short-circuit at the apical end to impose the condition at the helicotrema: that *p*_*N*_ is equal to zero. Although the elemental approach reduces to the transmission line model, which provides considerable insight from an electrical engineering perspective under these restrictive assumptions, it should again be emphasised that it is not necessary to make these assumptions for the elemental approach to be valid, as discussed below. The elemental model should thus be seen as a generalisation of the transmission line approach.

### 2.5 Example of an elemental model of the human cochlea

The human cochlea is chosen as an example to illustrate the elemental approach with the parameters listed in Table 1. The choice of the number of elements used in the model, *N*, is a trade-off between accuracy and computation time. A useful rule-of-thumb, mentioned above, is that there should be at least six elements per wavelength. Assuming that the shortest wavelength associated with the cochlea motion is about 0.5 mm, this guideline suggests that the length of an element, Δ, should be less than about 80 μm, so that there would be a total of about 420 elements along the 35 mm length of the human cochlea. The simulations below have been run with *N* = 512 elements, but the magnitude of the response at all frequencies is changed by less than 0.1 dB if twice this number of elements are used, suggesting that 512 elements is an appropriate choice. The coupled BM response of a uniform model with 1D fluid dynamics has been calculated in the spatial domain at a given frequency and in the frequency domain at a given position, as shown in Fig. 3. This elemental approach, however, is capable of modelling more complicated cases, for example, taking cross-sectional area variation and 3D fluid dynamics into account, as discussed later in Section 3. The coupled BM responses are also shown in Fig. 3, for comparison, when both area non-uniformity, assumed to be linearly tapered (Ni et al., 2017), and 3D fluid dynamics are considered. The linear variation in the physical dimensions of the fluid chambers along the length of the cochlea is based on Thorne et al. (1999), and the average values of those at the two ends are used for the uniform model. The modelling parameters are listed in Table 1 and example code can be found in supplementary material.

**Table 1:** Assumed parameters of the elemental passive model for the human cochlea.

Variable	Symbol	Value
Length (mm)	*L*	35
Cochlear partition width at the base (mm)	*W*_*B*_	1.14
Cochlear partition width at the apex (mm)	*W*_*A*_	0.67
BM width at the base (mm)	*B*_B_	0.15 (Wever, 1949)
BM width at the apex (mm)	*B*_A_	0.50 (Wever, 1949)
Frequency at the base (kHz)	*f*_B_	20
Characteristic length for frequency variation (mm)	*l*	7
BM Q factor	*Q*	2.5
Cochlear fluid density (kg m^−3^)	*ρ*	1000
BM mass per area (kg m^−2^)	*m*_0_	0.28 (1D fluid coupling) 0.05 (3D fluid coupling)
Scala area at base (mm^2^)	*A*_1_	1.29
Scala area at apex (mm^2^)	*A*_2_	0.45
Average scala area (mm^2^)	*A*_0_	0.84
Number of discrete BM	*N*	512

## 3 More general forms of fluid coupling

**Fig. 4:**
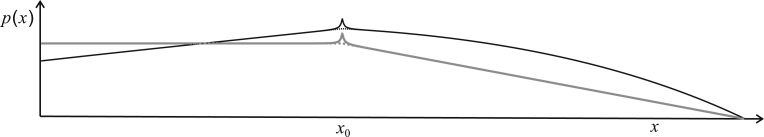
The pressure distribution along a uniform (grey lines) and non-uniform (black lines) cochlea due to excitation by the vibration of a single BM element, at *x*_0_ in the case of 1D fluid coupling (dashed) and 3D fluid coupling (solid). In this example, the human cochlear geometrical data for the non-uniform model is the same as that used in Ni et al. (2017). MATLAB Codes for generating the figure can be found in supplementary material.

Although a box shape with uniform cross-sectional area is widely used for modelling the cochlear mechanics, it is clearly a very simplified representation of the real cochlea geometry. The 1D, far-field, fluid dynamics, however, depend largely on the cross-sectional area of the chambers. On the other hand, the 1D representation will be invalid close to the characteristic place, where the wavelength of the BM motion becomes comparable with the chamber height. It is thus sensible to represent the fluid coupling more generally, to allow the geometrical variations and the 3D fluid dynamics to be taken into account. This section summarises procedures of incorporating cochlear geometrical variations, as well as the 3D fluid coupling. MATLAB codes for calculating the generalised fluid dynamics can be found in the supplementary material, together with an example of the non-uniform human cochlea model. Readers can also use their self-derived geometrical data for further investigations. Fig. 4 includes the results for both the non-uniform areas and 3D fluid coupling.

### 3.1 Non-uniform scala areas

It has been assumed above that the two fluid chambers have a uniform area along the length. This restriction can be removed by modifying equation (10) with incorporation of geometrical variations of the scala cross-sectional area, *A*(*x*), and the BM and cochlear partition width, *B*(*x*) and *W*(*x*) respectively (Ni et al., 2017). Generalised solutions to the 1D fluid dynamics in a tapered cochlear model have been proposed and discussed by Shera et al. (2004) using the Green's functions and by Ni et al. (2017) using the elemental method. If only a single BM element is excited by a velocity of *v*_0_, from *x*_0_−*Δ* to *x*_0_ and zero elsewhere, and assuming the pressure difference cross the BM is zero at *x* = *L,* account for the helicotrema, the 1D, far-field, fluid dynamics can be given as

(30) $${p_{\text{F}}\left(x\right)|}_{0<x<x_{0}-\Delta }=-\frac{16i\omega \rho \Delta^{2}v_{0}}{\pi^{2}}\sqrt{\frac{W\left(x_{0}\right)B\left(x_{0}\right)B\left(x\right)}{W\left(x\right)}}\int_{x_{0}}^{L}\frac{1}{A_{\text{e}}\left(x'\right)}dx^{\prime }\text{,}$$

(31) $${p_{\text{F}}\left(x\right)|}_{x_{0}<x<L}=-\frac{16i\omega \rho \Delta^{2}v_{0}}{\pi^{2}}\sqrt{\frac{W\left(x_{0}\right)B\left(x_{0}\right)B\left(x\right)}{W\left(x\right)}}\int_{x}^{L}\frac{1}{A_{\text{e}}\left(x^{\prime }\right)}dx^{\prime }\text{,}$$

where x' is dummy integration variable, *A*_e_(*x*) is the effective area, given by the harmonic mean of the upper and lower areas of the fluid chambers. It should be noted that equation (30) is for the pressure distribution basal to the excitation point and equation (31) is for the pressure distribution from the excitation point to the apex, as derived with more details in Ni et al. (2017). The lack of symmetry in the equations can be understood by considering the case where the effective area is constant along the cochlea, as in Elliott et al. (2011) for example, in which case the pressure due to an excitation at *x*_0_ gives rise to a far-field pressure that is constant basal to this point, since the fluid is uniformly accelerated in this region. The integral in equation (30) must thus be a constant in this case and does not depend on *x*. In contrast, the pressure difference apical to *x*_0_ in this simplified (uniform) case falls away linearly with *x*, until it goes to zero at *x* = *L*. Thus the integral in equation (31) must range from *x* to *L* to give such a variation in pressure.

If the areas of the fluid chambers in the cochlear models are divided up into *N* discrete sections, as for the BM, the integrals in equations (30), (31) can be approximated by summations to give the pressure at the *n*-th element as

(32) $${p_{\text{F}}\left(n\right)|}_{0<n<n_{0}-1}=-\frac{16i\omega \rho \Delta^{2}v_{0}}{\pi^{2}}\sqrt{\frac{W\left(n_{0}\right)B\left(n_{0}\right)B\left(n\right)}{W\left(n\right)}}\sum_{n^{\prime }=n_{0}}^{N}\frac{1}{A_{\text{e}}\left(n^{\prime }\right)}\text{,}$$

(33) $${p_{\text{F}}\left(n\right)|}_{n_{0}<n<N}=-\frac{16i\omega \rho \Delta^{2}v_{0}}{\pi^{2}}\sqrt{\frac{W\left(n_{0}\right)B\left(n_{0}\right)B\left(n\right)}{W\left(n\right)}}\sum_{n^{\prime }=n}^{N}\frac{1}{A_{\text{e}}\left(n^{\prime }\right)}\text{,}$$

where *n*_0_ = *x*_0_/Δ.

### 3.2 3D fluid dynamics

The vector of pressures in the elemental model is related to the vector of velocities by the fluid coupling matrix, equation (5). The physical significance of the columns of the matrix **Z**_FC_ in this equation are that they give the pressure distribution along the cochlear due to a single element of the BM vibrating, with all the other elements being fixed. If **Z**_FC_ is calculated assuming 1D fluid coupling in a uniform cochlear, so that **Z**_FC_ is given by equation (17), then this pressure distribution is given by the grey dashed line in Fig. 4. As noted above, the fluid is uniformly accelerated along the chambers on the basal side of the excitation point, generating a uniform pressure difference, but this pressure falls off linearly on the apical side of the excitation point until it goes to zero at the helicotrema.

1D fluid coupling assumes that the pressure is uniform across the cross-section of the cochlea, but as the wavelength of the slow wave becomes smaller there are significant variations of the pressure close to the BM, in the cross-section of the fluid chambers, and the fluid coupling is said to be 3D. The classic analysis of fluid coupling in this case, by Steele and Taber (1979a), uses a wavenumber analysis, as reviewed for the elemental model by Elliott et al. (2011). The additional component of the pressure due to its 3D nature is known as the near-field and when the fluid is excited by a single element, as above, this extends both across the cross-section of the fluid chamber and along the length of the cochlea. A great advantage of the elemental model is that it is only the longitudinal variation of the near-field pressure needs to be taken into account. This longitudinal variation for 3D fluid coupling is also shown, as the grey solid line, in Fig. 4, as calculated using the formulation of Elliott et al. (2011), and it is only significantly different from the 1D fluid coupling close to the point of excitation. The near-field pressure is the difference between these two cases and its magnitude and longitudinal distribution will depend on the local geometry around the BM. Since the near-field does not generally extend to the boundaries of the fluid chambers, however, it does not depend significantly on their cross-sectional shape, which considerably simplifies the modelling of the fluid coupling.

The factors that have the greatest effect on the near-field pressure are the width and the position of the BM across the cochlear partition, which separates the main two fluid chambers. It is shown in Elliott et al. (2011) and Ni and Elliott (2015) that to a good approximation the near-field pressure distribution due to the vibration of a single element is a decaying exponential function

(34) $$\begin{array}{c} p_{\text{NF}}\left(x\right)=i\omega \rho v_{0}\Delta \frac{8\left(a-b\right)B}{\pi^{2}\sqrt{b}W}e^{-\left|x-x_{0}\right|/\sqrt{b}W}\\ ={\hat{p}}_{\text{NF}}e^{-\left|x-x_{0}\right|/d}\text{,} \end{array}$$

where *x*_0_ is the position of vibrating element, *ω* is angular frequency, *v*_0_ is the linear velocity of the BM element, Δ is its width, *B* is the width of the BM, *W* is the width of the cochlear partition, and *a* and *b* are fitting coefficients (Ni and Elliott, 2015). Assuming that the BM begins from one side of the cochlear partition, the spatial ligament side, and that *B*/*W* is about 0.3, the peak near field pressure can be expressed as approximately,

(35) $${\hat{p}}_{\text{NF}}\approx 2i\omega \rho v_{0}\Delta \text{,}$$

where the factor of 2 is a parameter calculated based on fitted values of *a* and *b* (Ni and Elliott, 2015). Similarly, the decay distance, $d\approx \sqrt{b}W$, is approximately equal to

(36) d≈0.12W,

where the factor of 0.12 is another fitted parameter. The fluid coupling impedance in the case of 3D fluid coupling, $Z_{\text{FC}}^{3\text{D}}$, may thus be readily calculated by adding the discrete form of the equation above to the columns of the fluid coupling matrix calculated assuming 1D fluid coupling, $Z_{\text{FC}}^{1\text{D}}$, in Section 2.1, so that

(37) $$Z_{\text{FC}}^{3\text{D}}=Z_{\text{FC}}^{1\text{D}}+Z_{\text{FC}}^{\text{NF}}\text{,}$$

where the *n*-th column of $Z_{\text{FC}}^{\text{NF}}$ has the elements

(38) $$Z_{\text{FC}}^{\text{NF}}\left(n\right)\approx 2i\omega \rho \Delta e^{-\left|n-n_{0}\right|\Delta /0.12W}\text{.}$$

In fact, the 3D fluid coupling analysis has already been used in the definition of the effective height of the fluid chambers in the 1D case (Elliott et al., 2011). There is, however, only a small difference between the distribution of BM velocity along the cochlear predicted for a passive model with either 1D or 3D fluid coupling, as shown for example in Fig. 8 of Elliott et al. (2011). This comparison assumes that the mass of the BM in the 1D case is increased to account for the fluid loading. This increases the BM mass from about 0.05 kg/m^2^ in the 3D case, corresponding to a physically reasonable thickness for the organ of Corti of 50 μm, to a value of about 0.3 kg/m^2^ in the 1D case, since there is effectively a layer of fluid of total thickness about 250 μm that moves with the BM in this case and has to be accounted for in the micromechanics rather than the fluid coupling (Neely, 1981, Elliott et al., 2011).

## 4 Non locally-reacting micromechanics

The assumption made in Section 2.2 above is that the BM velocity at one position along the cochlea depends only on the pressure difference at that same point, i.e. it is locally reacting. There are, however, a number of mechanisms for longitudinal coupling along the BM, which will give rise to non locally-reacting behaviour, and this may be readily incorporated into the element model. The effect of these complexities in the model on its predicted response will depend on the extent of the assumed longitudinal coupling and the magnitude of the BM admittance. If reasonable values are assumed for the longitudinal coupling it is found that it again makes little difference to the predicted response in the passive cochlea, but would make a greater difference in a fully active one.

**Fig. 5:**
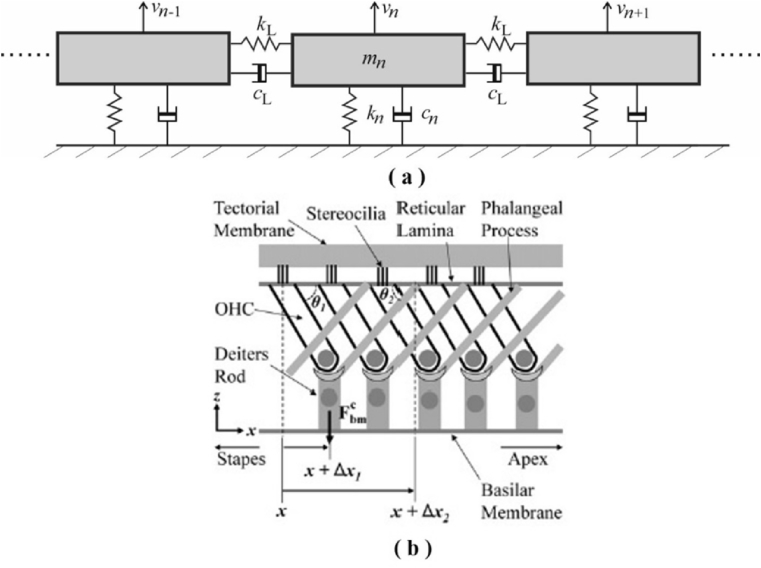
Longitudinal coupling along the cochlea represented by stiffnesses, *k*_L_, and dampings, *c*_L_, between adjacent elements (a) and the feedforward connection of the OHC (b) (Reprinted from Fig. 1 (a) in Yoon et al. (2011) with permission).

The first case of longitudinal coupling along the BM that we consider is due to its orthotropic behaviour, i.e. although its stiffness in the longitudinal direction is less than that in the radial direction, this longitudinal stiffness is not negligible (Emadi et al., 2004, Meaud and Grosh, 2010, Naidu and Mountain, 2001). Other authors have also emphasised the role of longitudinal damping along the BM (Chan and Yoon, 2015, Fisher et al., 2012, Reichenbach and Hudspeth, 2014). The simplest method of accounting for such behaviour in the elemental model is assuming that adjacent BM elements are coupled by a longitudinal stiffness, *k*_L_, and damping *c*_L_, as shown in Fig. 5.

In order to incorporate these elements into the BM admittance matrix, we first write down the equation for the pressure difference on the *n*-th element, from Fig. 5(a), as

(39) $$p_{n}=-\left(i\omega m_{n}+c_{n}+\frac{1}{i\omega k_{n}}\right)v_{n}-\left(c_{\text{L}}+\frac{1}{i\omega k_{\text{L}}}\right)\left(v_{n}-v_{n-1}\right)-\left(c_{\text{L}}+\frac{1}{i\omega k_{\text{L}}}\right)\left(v_{n}-v_{n+1}\right)\text{,}$$

where *m*_n_, *k*_n_ and *c*_n_ are the locally-reacting mas, stiffness and damping of the *n*-th BM element, assumed to be passive here for convenience, and *k*_L_ and *c*_L_ are the longitudinal coupling stiffness and damping terms. This equation can be written as

(40) $$p_{n}=-Z_{\text{BM}}\left(n,n-1\right)v_{n-1}-Z_{\text{BM}}\left(n,n\right)v_{n}-Z_{\text{BM}}\left(n,n-1\right)v_{n+1}\text{,}$$

where $Z_{\text{BM}}\left(n,n\right)=i\omega m_{n}+c_{n}+2c_{\text{L}}+\frac{1}{i\omega k_{n}}+\frac{2}{i\omega k_{\text{L}}}$ and $Z_{\text{BM}}\left(n,n-1\right)=Z_{\text{BM}}\left(n,n+1\right)$$=-c_{\text{L}}-\frac{1}{i\omega k_{\text{L}}}$.

**Fig. 6:**
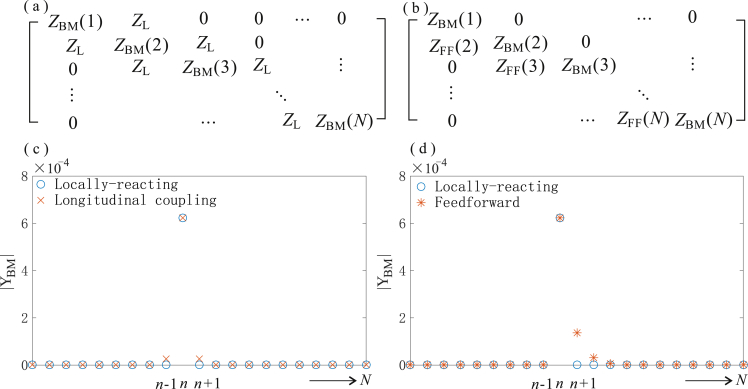
The structure of the BM impedance matrix in the case of symmetrical longitudinal coupling (a) as illustrated in Fig. 5 (a), and feedforward action in the organ of Corti (b), as illustrated in Fig. 5 (b), together with the longitudinal distribution of BM admittance corresponding to the velocity distribution along the cochlea when forced at a single point, given by a column of **Y**_BM_ = **Z**_BM_^−1^. In the longitudinal coupling case, *Z*_L_ is assumed to be 10% of *Z*_BM_ at each location, whereas a value of 50% is assumed for the feedforward case to more clearly show the effect.

A tri-diagonal matrix of BM impedances can thus be assembled from these elements as illustrated in Fig. 6(a), so that

(41) $$p=-Z_{\text{BM}}v\text{,}$$

in which case, assuming **Z**_BM_ is not singular, the BM admittance matrix required in the elemental model can be obtained by inverting **Z**_BM_. The resulting form of the BM admittance matrix is shown in Fig. 6(c).

Another form of longitudinal coupling that exists within the organ of Corti is illustrated in Fig. 5 (b) and is due to the way that the bottom of each outer hair cell, OHC, is attached to both to the BM by the Deiter's cell but also to more apical position along the reticular lamina by the phalangeal process (Fukazawa, 2002, Geisler and Sang, 1995, Steele et al., 1993, Yoon et al., 2011). Several models for this “feedforward” behaviour have been proposed, but one of the simplest was suggested by de Boer (2007), as a similar modification to the BM impedance model used for longitudinal coupling above, but acting only in one direction, so that

(42) $$p\left(x\right)=-Z_{\text{BM}}\left(x\right)v\left(x\right)-Z_{\text{FF}}\left(x\right)v\left(x-\Delta x_{3}\right)\text{,}$$

where Δ*x*_3_ is the longitudinal length scale over which the phalangeal process acts which is equal to Δ*x*_2_–Δ*x*_1_, in Fig. 5 (b). Although this feedforward distance varies from species to species and in one species varies along the length of the cochlea (Yoon et al., 2011), it is typically about 20 μm. This is much less than a wavelength of the slow wave, even in an active cochlea and so significantly less than the spatial discretisation that would normally be used in an elemental model. If a larger number of elements were used, so as to incorporate this feedforward effect, so that *L*/*N* = Δ*x*_3_, then equation (42) can be written in discrete form as

(43) $$p\left(n\right)=-Z_{\text{BM}}\left(n\right)v\left(n\right)-Z_{\text{FF}}\left(n\right)v\left(n-1\right)\text{.}$$

The impedance matrix now takes the form shown in Fig. 6(b) and the columns of the BM admittance matrix take the form shown in Fig. 6(d). In order to provide an illustration of this formulation, however, simulation have again been performed with 512 elements and it has been assumed, rather arbitrarily, that *k*_L_ and *c*_L_ are 10% of *k*_n_ and *c*_n_ for longitudinal coupling in equation (36) and $Z_{\text{FF}}\left(n\right)$ has a similar form to $Z_{\text{BM}}\left(n,n+1\right)$, but where *k*_L_ and *c*_L_ are 50% of *k*_n_ and *c*_n_ for feedforward coupling in equation (42).

The magnitude of the BM velocity along the cochlea in response to a pressure acting one only the *n*-th element, denoted |*Y*_BM_| in Fig. 6 (c) and (d), is clearly restricted to the *n*-th element when the BM is locally reacting, as expected, but spreads beyond this point in both directions if longitudinal coupling is assumed in the stiffness and damping, Fig. 6 (c), and spreads only in the apical direction if a feedforward model is assumed, Fig. 6 (d).

## 5 Time domain formulation

The elemental model can also be formulated in the time domain, to give a state space description of the cochlear mechanics (Elliott et al., 2007). It is more convenient in this formulation to include the excitation vector at the stapes in the fluid coupling part of the equations. Noting that the matrix **F** defined above has entirely real and frequency-independent elements, we can thus write the fluid coupling as

(44) $$Fp\left(t\right)=\dot{v}\left(t\right)+{\dot{v}}_{\text{s}}\left(t\right)\text{,}$$

where **F** is exactly as above, **p**(*t*) is the vector of time histories of the *M* pressures in the elemental model, $\dot{v}$(*t*) is the vector of accelerations of the elements and ${\dot{v}}_{\text{s}}$(*t*) is the vector containing the stapes acceleration as the first element, with all the other zero.

The micromechanics of the *n*-th element, for *n* = 2 to *N*−1, are now described in terms of individual state variable models, as

(45) $$\begin{array}{l} {\dot{x}}_{n}\left(t\right)=A_{n}x_{n}\left(t\right)+B_{n}p_{n}\left(t\right)\\ v_{n}\left(t\right)=C_{n}x_{n}\left(t\right)\text{,} \end{array}$$

where **x**_*n*_(*t*) is the vector of state variables for the *n*-th element, which has a pressure difference **p**_*n*_(*t*) acting on it, resulting in a velocity **v**_*n*_(*t*).

For example, if the passive micromechanics are described by a single degree-of-freedom system as above, then there are two state variables per element, **x**_11_(*t*) and **x**_12_(*t*), corresponding to the displacement and velocity of the element, and the equations above can be written as

(46) $$\left[\begin{array}{c} {\dot{x}}_{11}\left(t\right)\\ {\dot{x}}_{12}\left(t\right) \end{array}\right]=\left[\begin{array}{cc} -c_{n}/m_{n} & -k_{n}/m_{n}\\ 1 & 0 \end{array}\right]\left[\begin{array}{c} x_{11}\left(t\right)\\ x_{12}\left(t\right) \end{array}\right]+\left[\begin{array}{c} \frac{1}{m_{n}}\\ 0 \end{array}\right]p_{n}\left(t\right)\text{,}$$

and $v_{n}\left(t\right)=\left[\begin{array}{cc} 0 & 1 \end{array}\right]\left[\begin{array}{c} x_{11}\left(t\right)\\ x_{12}\left(t\right) \end{array}\right]\text{.}$

The dynamics of the middle ear can be similarly formulated to relate *p*_1_(*t*) to *v*_1_(*t*).

The uncoupled micromechanics of all the elements can thus be gathered together in a combined matrix equation

(47) $$\begin{array}{l} \dot{x}\left(t\right)=A_{\text{E}}x\left(t\right)+B_{\text{E}}p\left(t\right)\\ v\left(t\right)=C_{\text{E}}x\left(t\right)\text{,} \end{array}$$

where

(48) $$\begin{array}{l} x^{\text{T}}\left(t\right)={\left[\begin{array}{cccc} x_{1}^{\text{T}}\left(t\right) & x_{2}^{\text{T}}\left(t\right) & \cdots & x_{M}^{\text{T}}\left(t\right) \end{array}\right]}^{\text{T}}\\ v^{\text{T}}\left(t\right)={\left[\begin{array}{cccc} v_{1}^{\text{T}}\left(t\right) & v_{2}^{\text{T}}\left(t\right) & \cdots & v_{M}^{\text{T}}\left(t\right) \end{array}\right]}^{\text{T}}\\ A_{\text{E}}=\left[\begin{array}{ccccc} A_{1} & 0 & & \cdots & \\ 0 & A_{2} & & & \\ \vdots & & \ddots & & \\ & & & A_{M-1} & \\ 0 & & \cdots & & 0 \end{array}\right],\;B_{\text{E}}=\left[\begin{array}{ccccc} B_{1} & 0 & & \cdots & \\ 0 & B_{2} & & & \\ \vdots & & \ddots & & \\ & & & B_{M-1} & \\ 0 & & \cdots & & 0 \end{array}\right]\text{.}\\ C_{\text{E}}=\left[\begin{array}{ccccc} C_{1} & 0 & & \cdots & \\ 0 & C_{2} & & & \\ \vdots & & \ddots & & \\ & & & C_{M-1} & \\ 0 & & \cdots & & 0 \end{array}\right] \end{array}$$

Substituting the expression for **p**(*t*) above into this and noting that $\dot{v}\left(t\right)=C_{\text{E}}\dot{x}\left(t\right)$. Since all the elements of **C**_E_ are real, the state space model for the coupled elemental model can be written as

(49) x(t)=Ax(t)+Bu(t),

where

(50) $$\begin{array}{l} A={\left[I-B_{E}F^{-1}C_{E}\right]}^{-1}A_{E}\\ B={\left[I-B_{E}F^{-1}C_{E}\right]}^{-1}B_{E}\\ u=F^{-1}{\dot{v}}_{s}\text{.} \end{array}$$

**Fig. 7:**
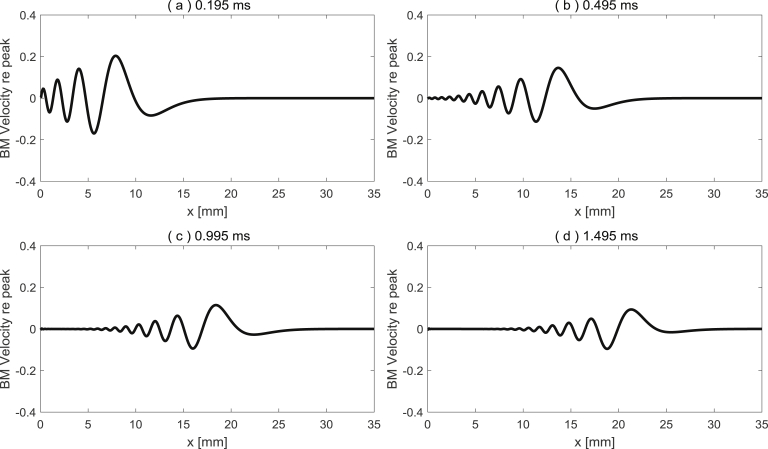
Examples of the instantaneous responses along the cochlea at different time instants when excited by an impulse of velocity at the stapes, (a) 0.195 ms, (b) 0.495 ms, (c) 0.995 ms and (d) 1.495 ms, using the state space model.

Again, an example MATLAB code can be found in the supplementary material, which formulates the elemental model in the time domain using a state space description (Elliott et al., 2007), and examples of the instantaneous responses along the cochlea, calculated at different time instants, when excited by an impulse of velocity at the stapes, are shown in Fig. 7. Readers could use this code as a template in order to incorporate different types of nonlinearity, for example, or to include modification to increase the computational efficiency (Pan et al., 2015).

## 6 Summary and conclusions

This paper describes an elemental approach to modelling the mechanics of the cochlea, particularly the interaction between the BM dynamics and the fluid coupling. The elemental model reduces to the classical transmission line formulation for a box model if the BM dynamics are locally-reacting and the fluid coupling is 1D, but is seen to be valid even without these restrictive assumptions. In particular, modifications are described for the model to incorporate non-uniform scala and BM dimensions, 3D fluid coupling and both symmetric and feedforward forms of longitudinal coupling along the BM.

Elemental formulations are described in the frequency domain, for linear models, and in the time domain, with the potential for extension to nonlinear models, and MATLAB programmes are included for these models in the supplementary material.

## Appendix A Supplementary data

mmc1 mmc1
