# Supplementary material for: An elemental approach to modelling the mechanics of the cochlea
Source: Hear Res. 2018 Mar;360:14–24. doi: 10.1016/j.heares.2017.10.013 (PMC5854296; doi:10.1016/j.heares.2017.10.013)
Supplement: mmc1 [file mmc1.zip › Guide to MATLAB example Code.doc]

**Guide to MATLAB Code**

By **Guangjian Ni**

[niguangjian@tju.edu.cn](mailto:niguangjian@tju.edu.cn)

October 2017

MATLAB scripts/functions are provided for illustrating the elemental approach and model used for modelling the cochlear mechanics, as described by Elliott and Ni in “**An elemental approach to modelling the mechanics of the cochlea**”, in the Special Issue of Hearing Research on “Computational Models in Hearing”. Most of the files should contain enough comments to help explain what each block does, but if there is anything confusing, please let me know.

The human cochlea is chosen for simulation here, but adaption of the code to other mammalian species is relatively straightforward, given the corresponding values of the variables in Table 1, as supplied, for example, in Ni et al (2017). There are two examples in the supplementary material: one for calculating the fluid coupling and the coupled response, and the other for providing a state space model for time domain simulations.

The main scripts and functions are listed below with example plots.

**1. Fluid coupling and coupled response**

**FluidCoupling_Human.m** is the main script for calculating the fluid coupling and the coupled response in the human cochlea. It generates results shown in Figure 2 and Figure 4. If you want to run the simulation for other species, you will need to create a file similar to **Human.mat**, which gives geometrical variations of the human cochlea.

**longwave_ZL.m** is a function that computes the fluid impedance due to the 1D, far-field, component. Details can be found in Ni et al. (2017).

**shortwave_ZS.m** is a function that the fluid impedance due to the 3D, near-field, component. Details can be found in Elliott et al. (2011).

**The BM response calculated using the elemental model of the human cochlea in the spatial domain (left column) and in the frequency domain (right column) with a uniform scala area and 1D fluid coupling, non-uniform scala area and 1D fluid coupling and non-uniform scala area and 3D fluid coupling.**

**The pressure distribution along a uniform (grey lines) and non-uniform (black lines) cochlea due to excitation by the vibration of a single BM element, at *x*_0_ in the case of 1D fluid coupling (dashed) and 3D fluid coupling (solid).**

2. **Time domain formulation**

**statespace_example.m** provides an example to formulate the elemental model in state space form and solve in the time domain using MATLAB ode45 solver. This example code provides three types of input, which are click, sinusoidal and user-defined vector.

**ss_formulation.m** is a function handle which will be called by ode45 solver.

**Examples of the instantaneous responses along the cochlea at different time instants when excited by an impulse of velocity at the stapes, (a) 0.195 ms, (b) 0.495 ms, (c) 0.995 ms and (d) 1.495 ms, using the state space model.**

**Examples of the instantaneous responses along the cochlea at different time instants when excited by a sinusoidal velocity at the stapes, (a) 0.195 ms, (b) 0.495 ms, (c) 0.995 ms and (d) 1.495 ms, using the state space model.**

**Examples of the instantaneous responses along the cochlea at different time instants when excited by user-defined velocities at the stapes, (a) 0.195 ms, (b) 0.495 ms, (c) 0.995 ms and (d) 1.495 ms, using the state space model.**

References:

Elliott, S. J., Lineton, B., and Ni, G. (2011). "Fluid coupling in a discrete model of cochlear mechanics," J. Acoust. Soc. Am. 130, 1441-1451.

Ni, G., Sun, L., and Elliott, S. J. (2017). "A linearly tapered box model of the cochlea," J. Acoust. Soc. Am. 141, 1793-1803.
